# *Chlamydia psittaci* PmpD-N Exacerbated Chicken Macrophage Function by Triggering Th2 Polarization and the TLR2/MyD88/NF-κB Signaling Pathway

**DOI:** 10.3390/ijms21062003

**Published:** 2020-03-15

**Authors:** Jun Chu, Xiaohui Li, Guanggang Qu, Yihui Wang, Qiang Li, Yongxia Guo, Lei Hou, Jue Liu, Francis O. Eko, Cheng He

**Affiliations:** 1 Key Lab of Animal Epidemiology and Zoonosis of the Ministry of Agriculture, College of Veterinary Medicine, China Agricultural University, Beijing 100193, China; tongtongchujun@163.com (J.C.); B20173050394@cau.edu.cn (X.L.); wyhairforce@126.com (Y.W.); liqiang5973@163.com (Q.L.); guoyongxiahh@gmail.com (Y.G.); 2 Beijing Key Laboratory for Prevention and Control of Infectious Diseases in Livestock and Poultry, Institute of Animal Husbandry and Veterinary Medicine, Beijing Academy of Agriculture and Forestry Sciences, Beijing 100097, China; hlbj09@163.com (L.H.); liujue@263.net (J.L.); 3 Shandong Binzhou Animal Science and Veterinary Medicine Academy, Binzhou 256600, China; quguanggang@aliyun.com; 4 Department of Microbiology, Biochemistry and Immunology, Morehouse School of Medicine, Atlanta, GA 30310, USA; feko@msm.edu

**Keywords:** *Chlamydia psittaci*, HD11 macrophage, PmpD-N, signaling pathway, Th2 immune response

## Abstract

The polymorphic membrane protein D (PmpD) is a highly conserved outer membrane protein which plays an important role in pathogenesis during *Chlamydia psittaci* infection. In this study, we evaluated the ability of the N-terminus of PmpD (PmpD-N) to modulate the functions of chicken macrophages and the signaling pathway(s) involved in PmpD-N-induced Toll-like receptors (TLRs), as well as interleukin (IL)-6 and IL-10 cytokine secretions. Thus, HD11 macrophages were treated with exogenous and intracellular PmpD-N of *C. psittaci*. The chlamydial growth was evaluated by enumeration of chlamydial loads in the infected macrophages. The phagocytic function of macrophages following PmpD-N treatment was detected by fluorescein-labeled *Escherichia coli* (*E. coli*). The concentration of nitric oxide (NO) secreted by HD11 macrophages was measured by the amount of NO_2_^-^ in the culture supernatant using the Griess method. The cytokine secretions were assessed using multiplex cytokine ELISA kits. Expression levels of TLRs, myeloid differentiation factor 88 (MyD88), and nuclear factor kappa B (NF-κB) were analyzed by a Western blotting assay, as well as a luciferase assay, while NF-κB p65 nuclear translocation was assessed by confocal microscopy. The nuclear translocation of the transcription factor NF-κB was confirmed by evaluating its ability to combine with the corresponding promoter using the electrophoretic mobility shift assay (EMSA). After treatment with exogenous or endogenous PmpD-N, chlamydial loads and phagocytic functions were reduced significantly compared with those of the plasmid vector group, while NO secretions were reduced significantly compared with those of the lipopolysaccharide (LPS) treatment. Stimulation of HD11 cells with PmpD-N provoked the secretion of the Th2 cytokines, IL-6, and IL-10 and upregulated the expression of TLR2, TLR4, MyD88, and NF-κB. Furthermore, inhibition of TLR2, MyD88, and NF-κB in HD11 cells significantly decreased IL-6 and IL-10 cytokine levels, while NO production and phagocytosis increased significantly, strongly suggesting their involvement in PmpD-N-induced Th2 cytokine secretion and macrophage dysfunction. Our data indicate that *C. psittaci* PmpD-N inhibited macrophage functions by activating the Th2 immune response and the TLR2/MyD88/NF-κB signaling pathway.

## 1. Introduction

*Chlamydia psittaci* is an obligate intracellular Gram-negative bacterium that can cause chlamydiosis, which is characterized by fever, chills, headache, dyspnea, and cough in psittacine birds, domestic poultry, as well as wild fowl [1]. Importantly, *C. psittaci* is also a zoonotic pathogen that can infect people who usually contact the droppings of infected birds via the inhalation of aerosols, causing pneumonia, encephalitis, endocarditis, increased risk of immunodeficiency virus infection, or even death [2]. Thus, avian chlamydiosis is not only associated with severe economic losses in the poultry industry but also with a potentially serious health hazard to humans [3,4].

Genome sequencing has revealed that the *pmp* gene encodes the largest membrane protein family—polymorphic membrane proteins (Pmps)—in *Chlamydia* species [5,6]. Polymorphic membrane protein D (PmpD), as a member of the Pmps subfamily, has a unique feature regarding the genus and representative [7]. The Pmps family, in all chlamydial genomes, suggests an important function in chlamydial biology [8]. The observed diversity in the number of allele genes and protein sequence sizes (90–190 kDa) and the expression levels within and across *Chlamydia* species also suggest that Pmps could be responsible for the observed differences in pathogenesis across *Chlamydia* species [9].

Establishing a chronic bacterial infection requires the active invasion of the host immune response. Under appropriate conditions in vivo, as well as in vitro, these pathogenic bacteria survive and grow in both nonprofessional (epithelial) [10,11] and professional (macrophage) phagocytic cells [12]. A major arm of the innate immune defense is constituted by macrophages, which fight infections by removing bacteria and triggering an adaptive immune response. However, some pathogenic *Chlamydia* infect, survive in macrophages as a vehicle, and then transfer to other host cells. At the end of their obligate intracellular development, *Chlamydia* are released into the extracellular environment, where they enter and multiply in neighboring epithelial cells [12].

Previous reports have described the bacterial invasion and host inflammation of PmpD [13,14,15,16]. Furthermore, recombinant PmpD has been suggested to function as an adhesin capable of inducing inflammatory cytokine production [17]. Both mouse and human cells infected with a *pmpD*-gene-modified strain showed that the PmpD protein is a virulence factor which plays an important role in the early days of the interaction between the pathogen and host [18]. In this study, we evaluated the ability of exogenous and endogenous PmpD N-terminal (PmpD-N) to modulate the immune functions of chicken macrophages and the signaling pathway(s) involved in PmpD-N-induced interleukin (IL)-6 and IL-10 cytokine secretions.

## 2. Results

### 2.1. Endotoxin Removal of the Recombinant PmpD-N and Construction of pEGFP-N1-pmpD-N Plasmids

**Figure 1:**
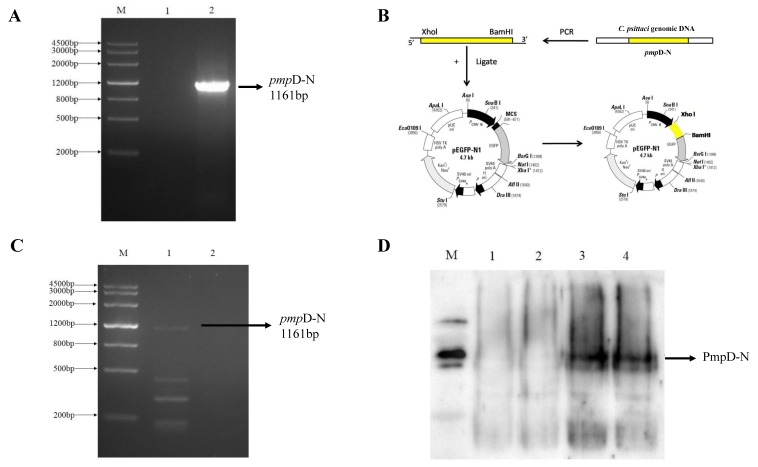
Construction of polymorphic membrane protein D N-terminal pEGFP-(*pmp*D-N) plasmids. (**A**) The *pmp*D-N gene was cloned and identified to product 116 bp by the PCR method. M: Marker (MD103); 1: Negative control; 2: *pmp*D-N gene. (**B**) Construction of pEGFP-N1-*pmp*D-N plasmids. (**C**) The *pmp*D-N genes were expressed in HD cells and identified by RT-PCR assay. M: Marker (MD103); 1: RNA product of pEGFP-N1-*pmp*D-N plasmid transfected into HD11 cells; 2: DNA product of pEGFP-N1 plasmid transfected into HD11 cells. (**D**) The PmpD-N proteins were detected by Western blotting assay.

The quantitative determination of endotoxins was 0.667 EU/mL compared with 40.3 EU/mL of the untreated recombinant PmpD-N proteins. The *pmp*D-N gene (GenBank ID: CP002549.1) was cloned (Figure 1A) and constructed successfully into the pEGFP-N1 vector (Figure 1B). Based on RT-PCR, the *pmp*D-N gene was confirmed to express in HD11 cells (Figure 1C), and PmpD-N expression was identified by a Western blotting assay to react with goat anti-rabbit HRP-conjugated antibody (Figure 1D).

### 2.2. The Effect of PmpD-N and pEGFP-N1-pmpD-N on the Replication of C. Psittaci in HD11 Cells

**Figure 2:**
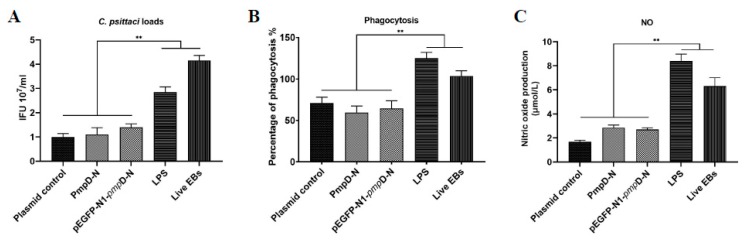
The effect of PmpD-N on chlamydial loads, phagocytic function, and nitric oxide (NO) production in the HD11 cells post-treated with PmpD-N or pEGFP-N1-pmpD-N. (**A**) Chlamydial loads were reduced in the PmpD-N and pEGFP-N1-pmpD-N groups compared with lipopolysaccharide (LPS), live EBs, or vector. (**B**) Phagocytic activities were reduced in the PmpD-N and pEGFP-N1-pmpD-Ngroups compared with LPS, live EBs, or vector. (**C**) NO production was reduced in the PmpD-N and pEGFP-N1-pmpD-Ngroups compared with LPS or live EBs (** *p* < 0.01 when compared with the live EBs group or the LPS group).

To evaluate the effect of PmpD-N and pEGFP-N1-*pmp*D-N plasmids on the replication of *C. psittaci* in HD11 cells, we calculated the inclusion forming units (IFUs) in HD11 cells after treatment with exogenous PmpD-N or the plasmids. After treatment with diverse concentrations of the PmpD-N (4–20 μg/mL), *C. psittaci* growth was determined and a moderate concentration (10 μg/mL) was used in the subsequent experiments (data not shown). The IFUs of *C. psittaci* in the PmpD-N- and pEGFP-N1-*pmp*D-N-treated HD11 cells were decreased significantly compared with the LPS and live EB control groups (*p* < 0.01) (Figure 2A).

### 2.3. The Effect of PmpD-N on the Phagocytic Function of HD11 Cells

To determine the effect of exogenous or intracellular PmpD-N on the phagocytic function of HD11 cells, we detected the phagocytic function after treatment with exogenous PmpD-N or the plasmids. The data showed that the phagocytic function (represented by the percentage of phagocytosis) of PmpD-N- or pEGFP-N1-*pmp*D-N-treated HD11 cells was reduced significantly (*p* < 0.01) compared with the LPS group or the live EB group. In contrast, the phagocytic function of live-EB-treated HD11 cells had significant changes compared with other groups (Figure 2B). However, no significant difference was found among the PmpD-N, the pEGFP-N1-pmpD-N, and the plasmid control groups.

### 2.4. The Effect of PmpD-N on the Release of Nitric Oxide in HD11 Cells

Activated macrophages can secrete immune-active nitric oxide, which can directly kill pathogenic microbes [19]. However, nitric oxide is a free radical, and its half-life in vivo is only about 30 s, so it is difficult to detect in the medium. To determine the effect of PmpD-N treatment on the release of nitric oxide in HD11 cells, the quantity of nitrites (NO2-) in the medium was measured and used as an indirect estimate of the nitric oxide concentration.

No significant difference of released nitric oxide was found among the PmpD-N, the pEGFP-N1-pmpD-N, and the plasmid control groups (*p* > 0.05).

In contrast, the release of nitric oxide in live-EB- or LPS-treated HD11 cells was increased significantly (*p* < 0.01) compared with the other groups (Figure 2C).

### 2.5. The Effect of PmpD-N on Cytokine Secretions in HD11 Cells

**Figure 3:**
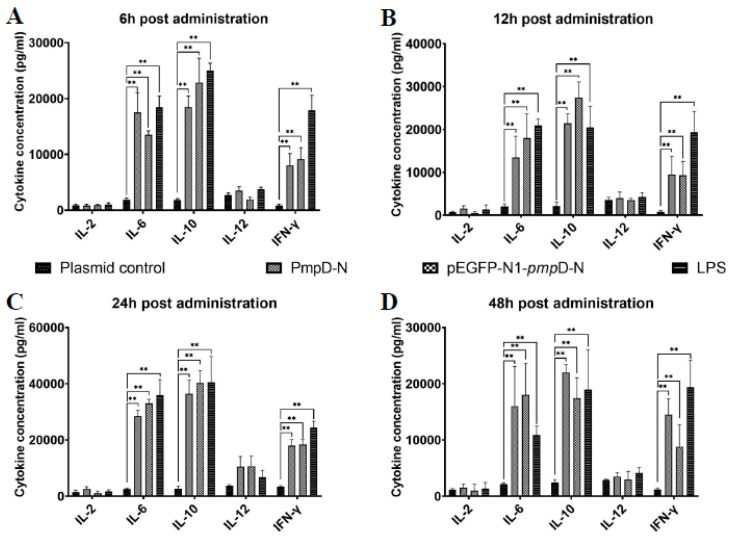
Cytokine secretions post-treatment with PmpD-N or pEGFP-N1-pmpD-N. At indicated time points after infection, the levels of cytokines (interleukin (IL)-2, IL-6, IL-10, IL-12, and IFN-γ) secreted were measured using chicken cytokine ELISA kits (**A**) 6h; (**B**).12h; (**C**).24h; (**D**).48h. Statistical analyses were performed with one-way analysis of variance (ANOVA) with Tukey’s post hoc multiple comparisons test (** *p* < 0.01 when compared with the plasmid control group, * *p* < 0.05 when compared with the plasmid control group).

To evaluate the effect of PmpD-N on the secretion of cytokines in HD11 cells, we detected the protein expression level of cytokines in HD11 cells after being treated with exogenous or intracellular PmpD-N. From 6 to 48 h, the expression levels of IL-6 and IL-10 increased significantly and amounted to the maximum at 24 h in the PmpD-N and pEGFP-N1-*pmp*D-N groups (*p* < 0.01) compared with the plasmid control group. However, IL-10 expression was not significantly different among the PmpD-N, intracellular PmpD-N, and LPS groups. On the contrary, IFN-γ expression was found to be less robust than that of IL-6 and IL-10. Compared with the LPS group, lower IFN-γ expression was observed in the PmpD-N and pEGFP-N1-*pmp*D-N groups at four time points. However, no significant difference was found between the PmpD-N and pEGFP-N1-*pmp*D-N groups. During all the observations, both IL-2 and IL-12 expression levels were not significantly different in all the groups (Figure 3).

### 2.6. The Effect of PmpD-N on the mRNA Expression of Toll-like Receptors (TLRs) and Myeloid Differentiation Factor 88 (MyD88) and Nuclear Factor kappa B (NF-κB) in HD11 Cells

We evaluated the effect of exogenous or intracellular PmpD-N on the mRNA expression level of different TLRs at different time points following *C. psittaci* infection. From 6 to 24 h, a higher mRNA expression level of TLR2 was found in the PmpD-N and pEGFP-N1-*pmp*D-N groups compared with the LPS and the plasmid control groups (*p* < 0.01). Moreover, the expression levels of TLR2 in stimulated HD11 cells were significantly higher than those of TLR1, TLR3, TLR4, and TLR5 at all time points. More interestingly, MyD88 in the stimulated HD11 cells was significantly higher than that of the plasmids group from 6 to 24 h (*p* < 0.01) and amounted to the maximum at 24 h poststimulation. Regarding TLR4 expression, LPS-stimulated HD11 cells expressed higher levels of the proteins as compared with the PmpD-N and pEGFP-N1-*pmp*D-N groups at all time points (*p* < 0.01).

**Figure 4:**
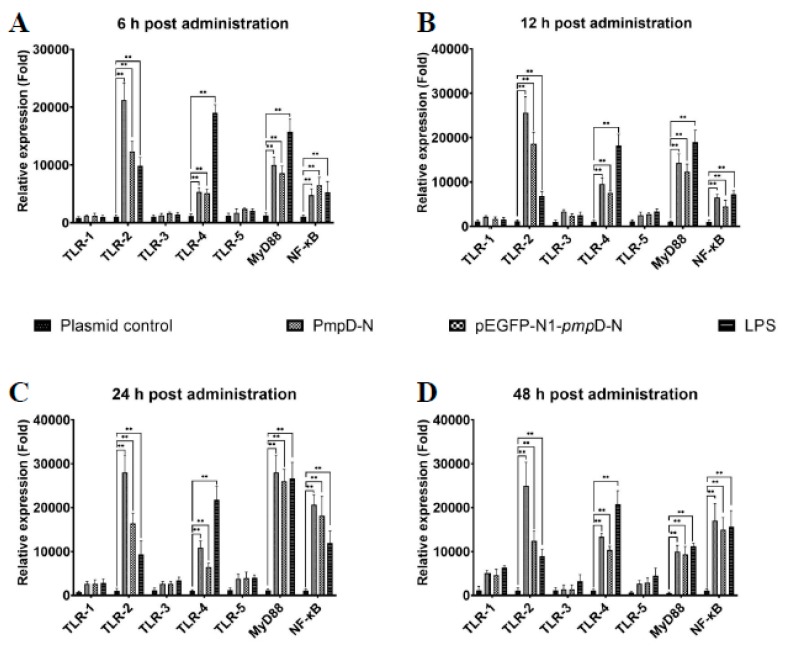
mRNA expression of Toll-like receptors (TLRs), myeloid differentiation factor 88 (MyD88), and nuclear factor kappa B (NF-κB) post-treatment with PmpD-N or pEGFP-N1-pmpD-N. The mRNA expression levels of TLR1–TLR5, MyD88, and NF-κB were determined by qRT- PCR. At different time points, the cell cultures were quantified in triplicate by qRT-PCR for levels of mRNA expression of TLRs and MyD88 by antigen-stimulated and *C.*-*psittaci*-infected HD11 cells (**A**) 6 h; (**B**).12 h; (**C**).24 h; (**D**).48 h. Statistical analyses were performed with one-way analysis of variance (ANOVA) with Tukey’s post hoc multiple comparisons test (** *p* < 0.01 when compared with the plasmid control group).

In addition, NF-κB expression was significantly increased in the PmpD-N group compared with the plasmid control group (*p* < 0.01) from 12 to 24 h, and no significant difference of NF-κB expression was found between the PmpD-N group and the pEGFP-N1-*pmp*D-N group at 24 h poststimulation (Figure 4).

### 2.7. The Effect of PmpD-N on the Nuclear Translocation of NF-κB in the Treated HD11

**Figure 5:**
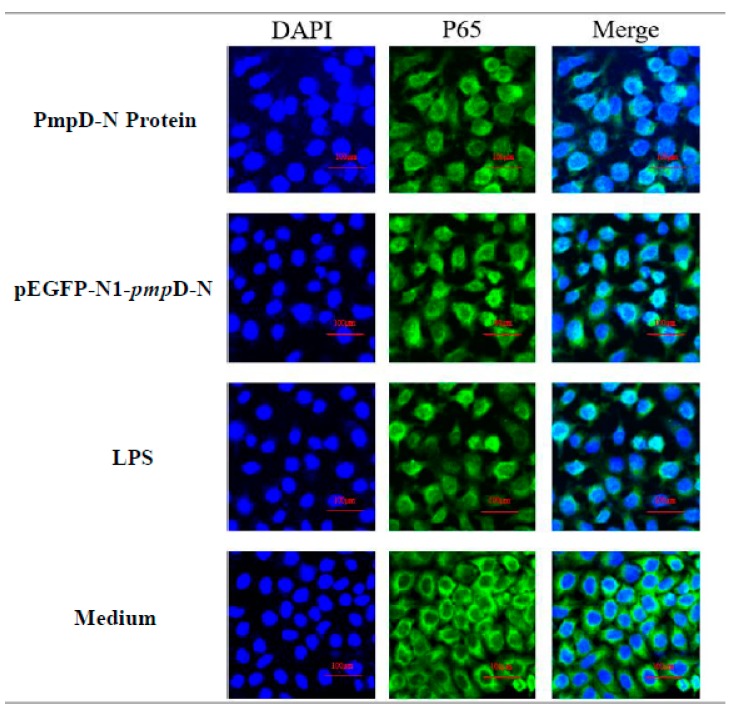
Detecting NF-κB p65 migration by confocal microscopy. NF-κB p65 nuclear migration in HD11 cells was detected by immunofluorescence microscopy. The p65 molecules migrated from the cytoplasm to the nucleus post-treatment with the recombinant PmpD-N, pEGFP-N1-pmpD-N, or plasmid control group.

Following the detection of NF-κB expression, we also evaluated the migration of NF-κB p65 to the nucleus post-treatment with PmpD-N and pEGFP-N1-*pmp*D-N using confocal microscopy. The nuclear NF-κB p65 subunit showed obvious translocation from the cytoplasm to the nucleus among the PmpD-N, pEGFP-N1-*pmp*D-N, and LPS groups. However, less migration of NF-κB p65 was found in the control group (Figure 5).

### 2.8. The Effect of PmpD-N on the Binding of the Transcription Factor NF-κB protein to the NF-κB Promoter DNA

**Figure 6:**
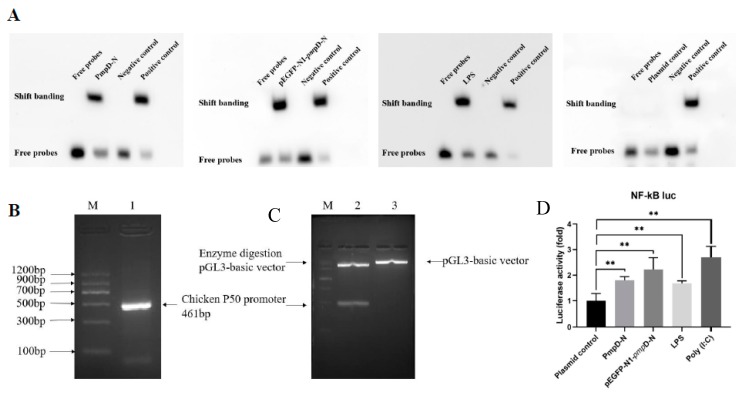
Binding and activation of transcription factor NF-κB with promoter. (**A**) NF-κB protein–oligonucleotide complexing was performed using a LightShift chemiluminescent electrophoretic mobility shift assay (EMSA). The transcription factor of NF-κB was bound to the promoter and showed band shifting post-treatment with rPmpD-N, pEGFP-N1-pmpD-N, or plasmid control. (**B**) Construction of chicken NF-κB transcription factor P50 promoters was identified to be positive by PCR. (**C**) The pGL3-basic-chp5-promoter was determined to be a 461 bp product by double enzyme digestion. M: Maker; 1: Chicken NF-κB transcription factor P50 promoters; 2: pGL3-basic-chp5-promoter; 3: pGL3-basic vector. (**D**) The NF-κB promoter activity was determined by luciferase activity in HD11 cells stimulated with exogenous or intracellular PmpD-N, LPS, or plasmid control for 6, 12, 24, and 48 h. Statistical analyses were performed with one-way analysis of variance (ANOVA) with Tukey’s post hoc multiple comparisons test. Statistically significant differences were evaluated at ** *p* < 0.01.

To determine the binding of NF-κB protein to the NF-κB-promoter-binding region, we analyzed the electrophoretic mobility shift assay (EMSA) of the NF-κB nuclear extract protein and NF-κB oligonucleotide probe (DNA) binding products of the different treatments in comparison with the control group. The results showed that the bands formed by the protein–DNA complexes of PmpD-N-, pEGFP-N1-*pmp*D-N-, and LPS-treated groups migrated much slower compared with the positive control group. In comparison with the plasmid control group, both PmpD-N and pEGFP-N1-*pmp*D-N induced considerable activity. However, no significant difference was found among the PmpD-N, pEGFP-N1-*pmp*D-N, and LPS groups (Figure 6A).

To determine the ability of PmpD-N to activate the NF-κB promoter activity, the construction of chicken NF-κB transcription factor P50 promoters was identified to be positive by PCR (Figure 6B), and the pGL3-basic-chp5-promoter was prepared to be a 461 bp product by double enzyme digestion (Figure 6C). Afterwards, HD11 cells were transfected with the pGL3-basic-NF-κB-promoter and the pRL-TK luciferase reporter for 12 h and then stimulated with poly (I:C), exogenous and intracellular PmpD-N, or LPS for 6, 12, 24, and 48 h. Luciferase activity, an indicator of NF-κB promoter activity, in each group was detected using the double luciferase reporter gene assay kit. The data showed that NF-κB promoter activity as determined by luciferase activity was significantly higher (*p* < 0.01) in HD11 cells stimulated with exogenous or intracellular PmpD-N or LPS compared with the plasmid control. The result indicated that PmpD-N activated the NF-κB promoter activity (Figure 6D).

### 2.9. The Effect of Short Interfering RNA (siRNA) or Treatment with NF-κB Inhibitor on Cytokine Secretion, Phagocytic Function, and NO Production

**Figure 7:**
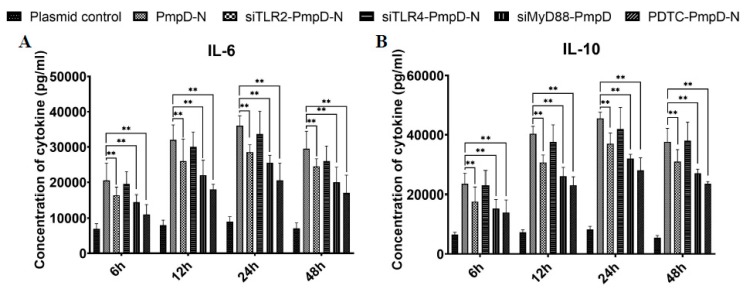
Effect of short interfering RNA (siRNA) or treatment with NF-κB inhibitor on cytokine secretions in HD11 cells. HD11 cells were transfected with siRNA targeting TLR2, TLR4, and MyD88 or treated with the NF-κB inhibitor pyrrolidine dithiocarbamate (PDTC) before stimulation with PmpD-N. Cell supernatants were collected and assayed for secretions of (**A**) IL-6 and (**B**) IL-10 using ELISA kits. Each experiment was repeated at least twice. Data are shown as the mean + SD values for triplicate cultures for one of two independent experiments. Statistically significant differences among different groups were evaluated with one-way analysis of variance (ANOVA) with Tukey’s post hoc multiple comparisons test and were considered to be statistically significant at ** *p* < 0.01.

We further investigated if inhibiting PmpD-N-mediated activation of TLR2, TLR4, MyD88, and NF-κB by using siRNA or an NF-κB inhibitor would influence the HD11 production of IL-6 and IL-10. The results from the cytokine ELISA analysis showed that targeting TLR2 and MyD88 with siRNA or inhibiting NF-κB with ammonium pyrrolidine dithiocarbamate (PDTC) resulted in a significant decrease (*p* < 0.01) in the magnitude of IL-6 and IL-10 cytokine secretion at 6, 12, 24, and 48 h post-treatment (Figure 7A,B).

**Figure 8:**
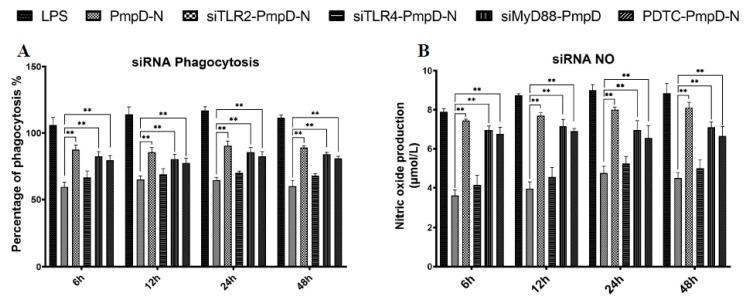
Effect of siRNA or treatment with the NF-κB inhibitor on phagocytic function and NO production in HD11 cells. HD11 cells were transfected with siRNA targeting TLR2, TLR4, and MyD88 or treated with the NF-κB inhibitor PDTC before stimulation with PmpD-N. Phagocytic (**A**) function and NO (**B**) production were determined in cell supernatants. Each experiment was repeated at least twice. Data are shown as the mean ± SD values for triplicate cultures for one of two independent experiments. Statistically significant differences among different groups were analyzed with one-way analysis of variance (ANOVA) with Tukey’s post hoc multiple comparisons test.

After treatment with siRNA targeting TLR2, TLR4, and MyD88, phagocytic capability and NO production were elevated significantly in the PmpD-N and pEGFP-N1-pmpD-N groups from 12 to 24 h, and no significant difference was observed at 48 h compared to those of the plasmid control group. Moreover, HD cells treated with PDTC displayed the same trends of phagocytic ability (Figure 8A) and NO production at the same time points (Figure 8B).

## 3. Materials and Methods

### 3.1. Quantitative Endotoxin of the Recombinant PmpD-N

The ELISA kits for chicken IL-2, IL-6, IL-10, IL-12, and IFN-γ were obtained from Kingfisher Biotech (St. Paul, MN, USA) and used according to the manufacturer’s instructions. TransZol Up Plus RNA kit (Catalog number: ER501–01) and TransScript One-Step gDNA Removal and cDNA Synthesis SuperMix (Catalog number: AT311–03) were purchased from TransGen Biotech (Beijing, China). SYBR Premix Ex Taq (2×) kit (Catalog number: RR420A) was obtained from Takara Bio (Dalian, China). Anti-TLR2 and -TLR4 antibodies were obtained from Santa Cruz Biotechnology (Shanghai, China). Anti-MyD88 was obtained from Sigma-Aldrich, and anti-NF-κB and anti-glutaraldehyde phosphate dehydrogenase (GAPDH) were from Cell Signaling (Danvers, MA, USA). The NF-κB inhibitor PDTC was purchased from Sigma-Aldrich (Catalog number: P6875) and diluted to a final concentration of 100 μM. Recombinant PmpD-N was purified and determined quantitatively as previously described [15] and supplied as lyophilized material at a concentration of 0.1 mg/mL. In order to remove enterotoxins from the recombinant proteins, the biomass was resuspended in NiNTA buffer A, sonicated, and clarified. Then, the supernatant was discarded, the pellet was resuspended in NiNTA (denoted buffer A) using an Ultra-Turrax T25 digital (IKA) with the dispersing element S25N-8G at 10,000–14,000 rpm for 30–60 s, and the qTP was added. Chromatography (AKTA™ avant 25, GE Healthcare, Shanghai, China) was run according to the manufacturer’s instructions as previously described [20]. The endotoxin level was determined using the EndoZyme^®^ II rFC Assay (Hyglos GmbH, Munich, Germany) according to the manufacturer’s instructions.

Construction of pEGFP-N1-*pmp*D-N plasmids and macrophage transfection. Firstly, the *pmpD-N* gene was amplified and cloned as previously described [15,16]. Briefly, the targeted gene was constructed with the pEGFP-N1 vector to make pEGFP-N1-*pmp*D-N plasmids and was identified by double enzyme digestion and sequence analysis (BGI, Beijing, China). After identifying positive pEGFP-N1-*pmp*D-N plasmids, co-transfection with HD11 followed the jetPPRIME protocol (Polyplustranfection Polyplus-transfectiou® SA, Illkirch,, France). The transfected plasmids were identified by RT-PCR, SDA-PAGE, and Western blotting assay.

### 3.2. Cell Culture and Bacterial Strain

The chicken monocyte/macrophage cell line HD11 cells were provided by Professor Jian Qiao (China Agriculture University, Beijing, China). The cells were cultured in Dulbecco’s modified Eagle’s minimal essential medium (DMEM) (Sigma-Aldrich Inc., Beijing, China), supplemented with 5% heat-inactivated fetal calf serum, 1% sodium pyruvate, 1% L-glutamine, and 0.5% gentamicin [21]. All products were obtained from Invitrogen (Invitrogen, Beijing, China). Cultures were incubated at 37 °C and 5% CO_2_. The *C. psittaci* 6BC strain (GenBank: CP002549.1) used in this study was kindly donated by Professor Yimo Wu (University of South China, Hunan, China) and was titrated as previously described [22].

### 3.3. The Culture of HD11 Cells with PmpD-N, pEGFP-N1-pmpD-N, or Plasmids

Chicken HD11 macrophages were cultured and incubated for 24 h at 37 °C until the cell density was up to 80%, then washed twice with sterile phosphate-buffered saline (PBS). The HD11 cells (5 × 10^5^ cells per well) were incubated with 10 μg/mL of the PmpD-N protein, the pEGFP-N1-*pmp*D-N plasmids, LPS, or live EB (positive control) or pEGFP-N1 plasmid (negative control) in a 24-well plate at 37 °C for 12 h. The cell-free supernatants were collected and used for determination of nitric oxide and cytokine concentration. The cells were processed for RNA extraction and fluorescent microsphere phagocytosis testing.

### 3.4. C. psittaci Infection of HD11 Cells Treated with PmpD-N, pEGFP-N1-pmpD-N, or Plasmids

HD11 cells were cultured as described above and concentrations of PmpD-N were independently optimized with 5, 10, 15, and 20 μg/mL. Chlamydial IFUs of 10 μg/mL of PmpD-N were chosen for this study. Afterwards, HD cells were stimulated with 10 μg/mL of PmpD-N or pEGFP-N1-*pmp*D-N plasmids for 1 h and then 500 μL of *C. psittaci* EB was added to each well at a MOI of 1. The LPS (Sigma-Aldrich Inc., Beijing, China) and vector were included as positive and negative controls, respectively. The treated cells were incubated in 5% CO_2_ at 37 °C for 1 h, the culture medium was dumped and they were washed with a fresh culture medium, and then incubated with a fresh medium for an additional 24 h.

## 4. Western Blotting Assay

Each protein extract (40 μg/well) was separated on a 12% SDS-PAGE and transferred to a 0.45 μm Immobilon-P PVDF membrane (Fisher Scientific, Ontario, Canada). After blocking with 5% (w/v) nonfat dried milk solution (Cell Signaling Technology, MA, USA) at room temperature for 1 h, membranes were incubated with primary antibodies against PmpD-N at 4 °C overnight. Membranes were then washed with PBS containing Tween 20 (PBST) and incubated with HRP-conjugated anti-rabbit secondary antibody (Thermo Fisher Scientific, Shanghai, China) at room temperature for 1 h. Proteins were detected with the Pierce ECL Western blotting substrate and visualized using a Tanon 5200 Imaging system (Tanon Science & Tech, Beijing, China).

### 4.1. The Effect of PmpD-N or pEGFP-N1-pmpD-N on the Phagocytic Function of Macrophages

HD11 cells were cultured in 6-well plates and stimulated with 10 μg/mL PmpD-N, pEGFP-N1-*pmp*D-N plasmids, LPS, or live EBs of *C. psittaci* at a MOI of 1 for 24 h. Then, the phagocytic function was detected with a Vybrant ^®^ Phagocytosis Assay kit (V6694, Thermo Fisher Scientific, IL, USA). The net phagocytic value and the net experimental value were calculated. The phagocytic activity was calculated using the following formula: Phagocytosis effect % = (net experimental value/net phagocytic value) × 100%.

### 4.2. Nitric Oxide Determination by C.-psittaci-Infected Macrophages

HD11 cells were stimulated with PmpD-N, pEGFP-N1-*pmp*D-N plasmids, LPS, or vector or live EBs of *C. psittaci* at a MOI of 1 for 24 h. The amount of nitric oxide produced in the culture supernatants of infected cells was estimated by measuring the concentration of nitrite (NO_2_^−^) using the Griess method. Specifically, culture supernatants (50 μL) were mixed with an equal volume of the Griess reagent (Beyotime Biotechnology, Shanghai, China) and incubated at room temperature for 10 min, and the absorbance of the reaction product was read on a Multiskan Mk III ELISA plate reader at 550 nm. Nitrite levels were calculated from a sodium nitrite (NaNO_2_) standard curve generated simultaneously from a 10-fold NaNO_2_ dilution series ranging from 320 to 0.3125 μM (in triplicate).

### 4.3. Cytokine and Toll-like Receptor Analysis

HD11 cells were stimulated with PmpD-N, pEGFP-N1-*pmp*D-N, LPS, or plasmids for 24 h. Then, total RNA was extracted from monolayers using the total RNA isolation reagent TransZol Up (TransGen Biotech, Beijing, China) at 1, 4, 8, and 12 h poststimulation. First-strand cDNA was transcribed using the TransGen Reverse Transcription kit (TransGen Biotech, Beijing, China). All protocols were performed according to the manufacturers’ instructions. Relative quantification of TLR1, TLR2, TLR3, TLR4, and TLR5 was performed using the SYBR Green PCR Master Mix kit (Takara, Dalian, China). The 2^−^^△△Ct^ method was used to calculate the relative gene expression levels of the samples. Gene expression was assayed quantitatively and normalized to GAPDH. The levels of cytokines secreted in HD11 cell culture supernatants were measured using chicken cytokine ELISA kits (Kingfisher Biotech, USA) essentially according to the manufacturer’s instructions.

### 4.4. Confocal Microscopy

HD11 cells were seeded in an 8-well microtiter plate (ibid, μ-Slide 8 Well #80826, Grafelfing Germany); treated with PmpD-N, pEGFP-N1-*pmp*D-N, LPS, or plasmids for 24 h; then fixed with 4% paraformaldehyde for 15 min at room temperature. After washing thrice with PBS, cells were incubated with 0.5% TritonX-100 for 15 min. Following an additional washing with PBS, cells were blocked with 1% BSA for 2 h and washed and incubated with anti-NF-κB p65 antibody for 1 h at room temperature. Cells were washed thrice with 1% BSA, incubated with Alexa Fluor^®^ 488 (Green-fluorescent dye Green) conjugated anti-rabbit IgG secondary antibody (Thermo Fisher Scientific, Shanghai, China) for 1 h in the dark, and counterstained with the nuclear stain DAPI (Blue). After a final wash, the cells were examined and photographed by immunofluorescence microscopy.

### 4.5. Electrophoretic Mobility Shift Assay (EMSA)

EMSA is a technology for studying the interaction between DNA-binding proteins and their associated DNA-binding sequences. The specific oligodeoxynucleotide duplex used as the DNA probe was a 5′-biotin-labeled NF-κB consensus sequence synthesized commercially (Beyotime Biotechnology, Shanghai, China). Detection of the NF-κB–oligonucleotide complex was performed using a LightShift chemiluminescent EMSA kit (Thermo Fisher Scientific, Shanghai, China) according to the manufacturer’s instructions. The shift binding was detected with the LightShift chemiluminescence detection reagents (Thermo Fisher Scientific, Shanghai, China) and images were acquired using an automated chemiluminescence image analyzer (Tanon Science & Tech, Beijing, China).

### 4.6. Luciferase Reporter Assay

HD11 cells were seeded in 24-well plates at a cell density of 4 × 10^4^ cells per well. After 16 h, the cells were transfected with chicken pGL3-basic-NF-κB-promoter plasmid and pRL-TK plasmid using the jetPRIME^®^ transfection reagent (Polyplus-transfectiou® SA, Illkirch, France, USA). The pRL-TK plasmid was used as an expression vector control. About 12 h after transfection, the cells were treated with poly (I:C) or 10 μg/mL PmpD-N, pEGFP-N1-*pmp*D-N, LPS, or plasmids for 12 h. Cell extracts were prepared and analyzed for luciferase activities using the dual-luciferase reporter assay kit (Promega Corporation, WI, USA) according to the manufacturer’s instructions.

### 4.7. Short Interfering RNA (siRNA)

siRNA targeting chicken TLR2 (sense 5′-GCUUAUUUACAGAUGCUACTT-3′, antisense 5′-GUAGCAUCUGUAAAUAAGCTT-3′), TLR4 (sense 5′-GCAGCCUUCCA-UGGCUUAATT-3′, antisense 5′-UUAAGCCAUGGAAGGCUGCTT-3′), and MyD88 (sense 5′-GCCAAAGACUUCAGAGCUGTT-3′, antisense 5′-CAGCUCUGAAGUCUU-UGGCTT-3′) were synthesized by a commercial company (Viewsolid Biotech, Beijing, China). HD11 cells were transiently transfected with the X-tremeGENE^TM^ siRNA Transfection Reagent (Roche Molecular Biochemicals, IN, USA) and 20 μM (the amount was optimized in preliminary experiments) siRNA targeting chicken TLR2, TLR4, or MyD88 for 24 h according to the manufacturer’s instructions. Briefly, 20 μM siRNA targeting chicken TLR2 or TLR4 for 24 h was incubated in each well at room temperature for 20 min, and HD11 cells were plated in the medium per well. All transfections were carried out in triplicate. Forty-eight hours after the addition of the siRNA, HD11 cells were treated with PmpD-N or plasmids for 24 h. Moreover, HD11 cells were pretreated with 100 μM of the NF-κB inhibitor PDTC (Merck, #5108–96-3 Merck KGaA, Darmstadt, Germany) for 1 h before stimulation with PmpD-N or plasmids. Cell supernatants were harvested after 24 h and assayed for IL-6 and IL-10 concentration by ELISA. Moreover, nitric oxide and phagocytic capacity in culture supernatants of infected cells were determined as described above.

## 5. Statistical Analysis

The data are presented as averages ± standard deviations (SDs), as indicated. Statistical comparisons were analyzed with one-way analysis of variance (ANOVA) with Tukey’s post hoc multiple comparisons test. Significant differences were determined at ** *p < 0.01* and statistically significant differences were determined at * *p* < 0.05. No statistical difference was denoted ns.

## 6. Discussion

Usually, *C. psittaci* causes severe systemic long-term latent infection in the host. As the main reservoir, highly infected ducks and pigeons threaten the poultry industry and human health. A more recent report indicates that birds inoculated with the high-virulence *C. psittaci* strain had suppressed immune response, leading to secondary infection of avian influenza virus H9N2 and high mortality in poultry [23]. Immune responses induced by *Chlamydia* infection in macrophages have previously been described, but the effect of polymorphic membrane proteins on macrophages at the adhesion stage with *C. psittaci* has not yet been investigated. Moreover, the effect of PmpD-N on immune response following *C. psittaci* infection is also unclear, hampering the development of a comprehensive control strategy against *C. psittaci* infection.

The initial finding of this study is that *C. psittaci* PmpD-N, a surface-exposed protein, influenced the early interaction of *C. psittaci* with the host cell. Both exogenous PmpD-N and intracellular PmpD-N might have inhibited chlamydial growth by limiting its uptake by HD11 cells. Subsequently, phagocytic activities were reduced significantly compared with those of the LPS group, indicating downregulation of the phagocytic ability of macrophages by PmpD-N proteins. This is the first report that immune evasion was triggered by PmpD-N of *C. psittaci* infection by inhibiting macrophage factions and attacking the first immune barriers. More importantly, this might shed light on understanding new control measures against avian chlamydiosis.

The decrease in the level of infection seen in HD11 cells could have been due to a decrease in the ability of HD11 cells to take up and internalize *C. psittaci* EBs triggered by exogenous or intracellular PmpD-N treatment. Moreover, the phagocytic functions of the exogenous PmpD-N protein group were significantly lower than those of the LPS and live EB groups. This confirmed again that both exogenous and intracellular PmpD-N can decrease the phagocytic ability of macrophages. This might explain postinfection macrophage dysfunction with different serotypes of *C. psittaci* in vitro [14]. The potential reasons for this might be due to the secretion of nitric oxide, binding specific receptors, chemokines, Th1/Th2 immune balance, and signaling pathways.

Nitric oxide (NO) has been documented in macrophages from human, horse, cow, goat, sheep, rat, mouse, and chicken. Nitric oxide was produced from L-arginine catalyzed by nitric oxide synthases (NOS) in vivo. Nitric oxide can act on its own cells or spread to neighboring cells, binding its receptor to, for example, transcription factors and proteases, which plays a series of regulatory roles [24,25,26]. Sustained production of NO induces macrophages with cytostatic or cytotoxic activity against viruses, bacteria, and tumor cells. In a previous report, macrophages activated by chlamydial infection upregulated the expression of NOS [27]. At 4 h post-treatment, the NO concentration in the HD11 supernatant was highly elevated for an infection with 10 μg/mL LPS or live EB instead of PmpD treatment. Moreover, poor phagocytic functions were observed in both exogenous and intracellular PmpD-N groups, which corresponded to lower chlamydial loads compared with the vector control group. This suggests that exogenous or intracellular PmpD-N had the capacity to quietly enter the macrophages, not stimulate NO secretions, and exacerbate the phagocytic capacity of infected macrophages; thus, the *Chlamydia* escaped macrophages to its killing effect. A previous report indicated NO secretions were dependent on infectious doses and time points. Increased NO production could be induced in macrophages with a moderate MOI dose and long-term treatment [27].

Previous studies have demonstrated that specific pattern recognition receptors (PRRs), such as TLRs, are stimulated upon recognition of various bacterial-pathogen-associated molecular patterns (PAMPs), leading to induction of immune responses mainly triggered via activation of the transcription factor [28,29]. Since HD11 expressed high levels of TLR2, MyD88, and NF-kB when stimulated with exogenous and intracellular PmpD-N, macrophage dysfunction and low clearance of *C. psittaci* were predominantly mediated by TLR2/MyD88/NF-κB. Our data are consistent with the previous report that *Chlamydia abortus* rPmp18.1 activated the expression of TLR4, MyD88, NF-κB p50, Caspase-1, and NF-κB p65 in treated dendritic cells (DCs) [30]. Chlamydial MIP lipoprotein can induce an inflammatory response in the pathogenesis of *Chlamydia trachomatis* via TLR2/TLR1/TLR6 and CD14 in human macrophages [31]. Additionally, binding of microbial PAMPs to TLRs can regulate the expression of genes that encode proinflammatory cytokines [27]. In the current study, higher expression levels of the Th2 cytokines IL-6 and IL-10 were observed compared with those of lower IFN-γ expression post-treatment with exogenous or intracellular PmpD-N, indicating that IL-6 and IL-10 secretions rely on signaling via TLR2/MyD88/NF-kB in macrophages. In order to verify the above data, we also investigated whether inhibiting PmpD-N-mediated activation of these proteins using siRNA would influence the HD11 production of IL-6 and IL-10. Furthermore, increasing phagocytic capability and high NO production were observed in the macrophages treated with siRNA targeting TLR2, TLR4, and NF-kB inhibitors. The results demonstrated that siRNA or an inhibitor targeting TLR2, MyD88, and NF-kB in HD11 cells significantly reduced IL-6 and IL-10 cytokine secretion, strongly suggesting the involvement of TLR2, MyD88, and NF-kB signaling in PmpD-N-induced IL-6 and IL-10 secretion. Our data indicate that PmpD-N might have an indirect influence on macrophage function via the TLR2/MyD88/NF-κB signaling pathway.

Recent reports indicate that IL-10 regulates innate immunity by directly acting on macrophages, DCs, and major histocompatibility complex class II proteins. IL-10 can also inhibit phagocytosis and microbial killing through limiting the production of reactive oxygen and nitrogen intermediates in response to IFN-γ. IL-10 limitation also resulted in the rapid elicitation of immune responses against *Chlamydia* and decreased levels of IL-10 correlated with protective anti-*Chlamydia* immunity [32]. In the present study, IL-10 and IL-6 expressions were dominant poststimulation, while a nonsignificant Th1-promoting response in infected HD11 cells was observed at 4 h poststimulation. In the study, PmpD-N-stimulated IFN-γ secretions were found to be less robust than those of IL-6 and IL-10 induced by the LPS group. Low IFN-γ levels might be associated with macrophage dysfunction, characterized as reduced NO production and poor phagocytic capacity. The results of cytokines showed that the Th2 system was activated during the interaction of PmpD-N and macrophages, which negatively impacted chlamydial clearance. In a previous report, specific pathogen free (SPF) chickens inoculated with a high-virulence *C. psittaci* strain were determined to have high IL-10 and IL-6 expressions, which resulted in immune suppression and secondary infection of H9N2 [23]. This has provided us with new evidence of *Chlamydia*-induced immune escape by PmpD-N proteins. These results are consistent with the observations of Beeckman et al. [25], as similar effects have also been observed for other intracellular pathogens such as *Mycobacterium tuberculosis*, *Leishmania major*, and *Histoplasma capsulatum* [33].

In conclusion, the results of the present study also indicate that *C. psittaci* PmpD-N reduces the immune function of macrophages, induces IL-6 and IL-10 secretion by TLR2 activation through the MyD88/NF-kB signaling pathways, and is thus beneficial for the immune escape of *Chlamydia psittaci*. This is the first explorative investigation of macrophage dysfunction mediated by *C.*-*psittaci*-specific PmpD-N. This study might shed light on drug development, molecular modification of vaccines, and the prevention and cure of *C. psittaci* infection.
