# *Correction:Chlamydia psittaci* PmpD-N Modulated Chicken Macrophage Function by Triggering Th2 Polarization and the TLR2/MyD88/NF-κB Signaling Pathway

**DOI:** 10.3390/ijms21072639

**Published:** 2020-04-10

**Authors:** Jun Chu, Xiaohui Li, Guanggang Qu, Yihui Wang, Qiang Li, Yongxia Guo, Lei Hou, Jue Liu, Francis O. Eko, Cheng He

**Affiliations:** 1 Key Lab of Animal Epidemiology and Zoonosis of the Ministry of Agriculture, College of Veterinary Medicine, China Agricultural University, Beijing 100193, China; tongtongchujun@163.com (J.C.); B20173050394@cau.edu.cn (X.L.); wyhairforce@126.com (Y.W.); liqiang5973@163.com (Q.L.); guoyongxiahh@gmail.com (Y.G.); 2 Beijing Key Laboratory for Prevention and Control of Infectious Diseases in Livestock and Poultry, Institute of Animal Husbandry and Veterinary Medicine, Beijing Academy of Agriculture and Forestry Sciences, Beijing 100097, China; hlbj09@163.com (L.H.); hlbj09@163.com (J.L.); 3 Shandong Binzhou Animal Science and Veterinary Medicine Academy, Binzhou 256600, China; quguanggang@aliyun.com; 4 Department of Microbiology, Biochemistry and Immunology, Morehouse School of Medicine, Atlanta, GA 30310, USA; feko@msm.edu

The authors would like to make the following corrections to their paper, published in the *International Journal of Molecular Sciences*. In the corrected version, we revised the title and figure legends in Figures 1, 2 and 5 due to indefinite expressions. As for funding resources, we deleted the NIH grant in light of it having no connection to the research. The following changes are acknowledged, and the changes do not affect the conclusions of the paper.

1. We changed a word in the title

*Chlamydia psittaci* PmpD-N modulated Chicken Macrophage Function by Triggering Th2 Polarization and the TLR2/MyD88/NF-κB Signaling Pathway.

2. We revised the figure legends of Figures 1, 2 and 5

The figure legends should be replaced with the following descriptions:

**Figure 1.** Construction of pEGFP-N1-*pmp*D-N plasmid and detection of PmpD-N expression. (**A**) The *pmp*D-N gene was amplified from genomic DNA of *C. psittaci* 6BC by PCR. M: Marker (MD103); 1: Negative control; 2: *pmp*D-N gene (1161 bp). (**B**) The amplified *pmp*D-N gene was inserted into the pEGFP-N1 vector to generate pEGFP-N1-*pmp*D-N. (**C**) The *pmp*D-N genes were expressed in HD cells and identified by RT-PCR assay. M: Marker (MD103); 1: DNA product of pEGFP-N1-*pmp*D-N plasmid transfected into HD11 cells; 2: RNA product of pEGFP-N1 plasmid transfected into HD11 cells. (**D**) The PmpD-N protein was detected by Western blotting analysis.

**Figure 2.** The effect of PmpD-N on chlamydial load, phagocytic function, and nitric oxide (NO) production in the HD11 cells after treatment with PmpD-N or pEGFP-N1-*pmp*D-N. (**A**) The data showed the mean number of chlamydial inclusion forming units (IFU) for each group. (**B**) Phagocytic activity was calculated as the ratio between the MFI of each group and the net phagocytic value. The data showed the percentage of phagocytic activity (±SD) for cultures for each experiment. (**C**) The concentration of NO was measured by the Griess method and differences were compared at ** *p* < 0.01.

**Figure 5.** Detection of NF-κB p65 migration by confocal microscopy. NF-κB p65 nuclear migration in HD11 cells was detected by immunofluorescence microscopy. The p65 unit was stained with Alexa Fluor^®^ 488(green) and nuclei were visualized by DAPI counterstain (blue). Note the diffuse distribution of NF-κB p65 (green) in the nucleus.

3. We revised the funding source as follows

